# Characterization of a novel swollenin from *Penicillium oxalicum* in facilitating enzymatic saccharification of cellulose

**DOI:** 10.1186/1472-6750-13-42

**Published:** 2013-05-20

**Authors:** Kang Kang, Shaowen Wang, Guohong Lai, Gang Liu, Miao Xing

**Affiliations:** 1 College of Life Science, Shenzhen Key Laboratory of Microbial Genetic Engineering, Shenzhen University, Shenzhen 518060, China; 2 College of Animal Science and Technology, Northwest A&F University, Yangling, Shaanxi 712100, China

**Keywords:** Cellulase, Cellulose, Expansin, Swollenin, *Penicillium oxalicum*, *Trichoderma reesei*

## Abstract

**Background:**

Plant expansins and fungal swollenin that can disrupt crystalline cellulose have great potential for applications in conversion of biomass. Recent studies have been mainly focused on *Trichoderma reesei* swollenin that show relatively low activity in the promotion of cellulosic hydrolysis. Our aim was to isolate a novel swollenin with greater disruptive activity, to establish an efficient way of producing recombinant swollenin, and to optimize the procedure using swollenin in facilitation of cellulosic hydrolysis.

**Results:**

A novel gene encoding a swollenin-like protein, POSWOI, was isolated from the filamentous fungus *Penicillium oxalicum* by Thermal Asymmetric Interlaced PCR (TAIL-PCR). It consisted of a family 1 carbohydrate-binding module (CBM1) followed by a linker connected to a family 45 endoglucanase-like domain. Using the cellobiohydrolase I promoter, recombinant POSWOI was efficiently produced in *T*. *reesei* with a yield of 105 mg/L, and showed significant disruptive activity on crystalline cellulose. Simultaneous reaction with both POSWOI and cellulases enhanced the hydrolysis of crystalline cellulose Avicel by approximately 50%. Using a POSWOI-pretreatment procedure, cellulases can produce nearly twice as many reducing sugars as without pretreatment. The mechanism by which POSWOI facilitates the saccharification of cellulose was also studied using a cellulase binding assay.

**Conclusion:**

We present a novel fungal swollenin with considerable disruptive activity on crystalline cellulose, and develop a better procedure for using swollenin in facilitating cellulosic hydrolysis. We thus provide a new approach for the effective bioconversion of cellulosic biomass.

## Background

Lignocellulose, consisting of cellulose, hemicellulose and lignin, is the most abundant and renewable resource that can be hydrolyzed into fermentable sugars to produce biofuel [1]. Natural cellulose is a linear polymer composed of thousands of glucose molecules linked via β-1-4-glycosidic bonds. It exists in highly ordered, tightly packed microfibrils, forming remarkable hindrance to cellulases [2-4]. Conversion of the crystalline regions of native cellulose into amorphous and accessible regions is a critical step, and is a great challenge for efficient hydrolysis of lignocellulose [5,6].

Expansins are a class of plant proteins that can promote the plant cell wall loosening by breaking down the hydrogen bonds among cellulose microfibrils or between cellulose fibers and the polysaccharide matrix [7-9]. Swollenin, a newly identified fungal protein with sequence similarity to plant expansins, is capable of weakening filter paper and disrupting the structure of cotton fiber and Valonia cell walls [10]. The disruptive activity of expansins and swollenin on plant cell walls can enhance the hydrolysis of biomass. While active and efficient heterologous production of expansins outside plants has not been reported, fungal swollenin has successfully achieved recombinant expression in several eukaryotic [11,12] and prokaryotic hosts [13]. Recent studies regarding swollenin have mainly focused on *T*. *reesei* SWOI [10,12-15]. Moreover, the effect of swollenin in the promotion of cellulosic hydrolysis has not been well investigated.

*Penicillium* species are another group of fungi capable of producing high activities of cellulase and hemicellulase [16-19]. They have higher β-glucosidase activity and better thermal stability of FPase activity than *Trichoderma* species [20,21]. To explore a swollenin with higher activity, we used *Penicillium oxalicum* for gene cloning of a novel swollenin. We present the disruptive activity of *P*. *oxalicum* swollenin on crystalline cellulose and its characteristics in enhancing cellulosic hydrolysis.

## Results

### *P*. *oxalicum* swollenin cloning and sequence analysis

**Figure 1:**
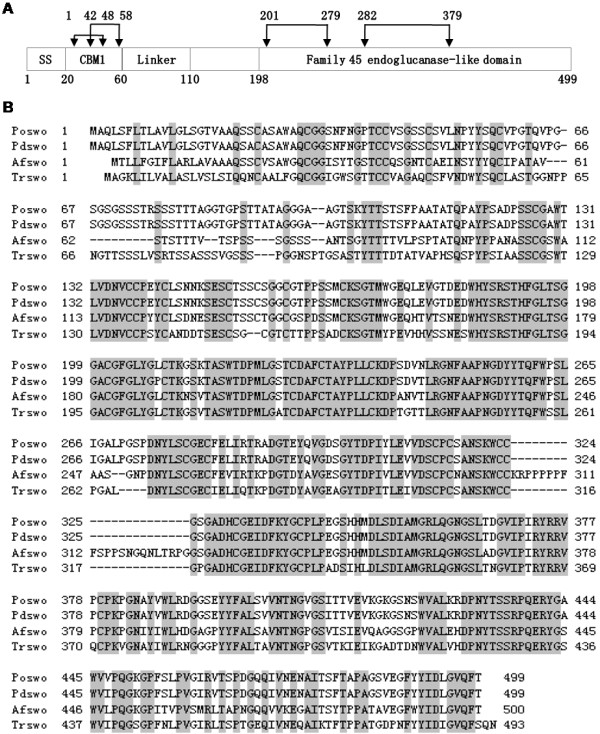
**Amino acid sequence analysis of POSWOI. A**: POSWOI consists of 20 amino acid signal sequences (SS), a family 1 carbohydrate-binding module (CBM1), a linker region and a family 45 endoglucanase-like domain. Double-headed arrows indicate the disulfide bonds. **B**: alignment of *P*. *oxalicum* swollenin (Poswo, GeneBank No. ADZ74267) with three other swollenins from *Trichoderma reesei* (Trswo, GeneBank No. CAB92328), *Penicillium decumbens* (Pdswo, GeneBank No. ACH57439), and *Aspergillus fumigatus* (Afswo, GeneBank No. XP_747748), respectively. Identical amino acids are shaded. Numbers show the position of amino acids.

Based on the alignment of published fungal swollenin sequences on Genebank database, a pair of degenerate primers was designed and used to successfully amplify a 210 bp swollenin fragment from *P*. *oxalicum* genomic DNA. The 5’ and 3’ flanking sequences of the isolated swollenin fragment were then obtained using the TAIL-PCR method. Finally, the full-length coding sequence of the *P*. *oxalicum* swollenin gene was amplified from genomic DNA, and termed *poswo1* (GeneBank No. HQ291307). Sequence analysis showed that *poswo1* contains 1,905 bps interrupted by six introns, and encodes 499 amino acids. Amino acid analysis indicated that POSWOI is homologous to other fungal swollenins (Figure 1B), and shows highly similarity (>99%) to the Penicillium decumbens swollenin, whose biochemical characteristics have not yet been reported. POSWOI has a predicted signal peptide of twenty amino acids at the N-terminal and four disulfide bonds (Figure 1A). Analysis using the online tool Prosite [22] shows that POSWOI consists of a family 1 carbohydrate-binding module (CBM1) followed by a linker connected to a family 45 endoglucanase-like domain (Figure 1A). Another tool, ProtParam, [23] revealed that the instability index and the estimated half-life of POSWOI is 38.09 and >20 h, respectively, suggesting the potential stability of POSWOI protein.

### Production of recombinant POSWOI

**Figure 2:**
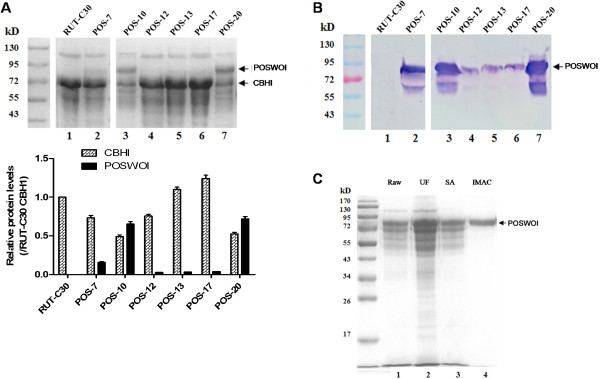
**Production and purification of recombinant POSWOI in *T. reesei *RUT-C30. A**: SDS-PAGE analysis of the culture supernatant of six *poswo1* transformants. Lane 1: parental strain *T*. *reesei* RUT-C30; Lane 2 ~ 7: *poswo1* transformants of POS-7, POS-10, POS-12, POS-13, POS-17, and POS-20. The quantity of the CBHI and POSWOI bands was evaluated by density scan. **B**: Western blot analysis of the same samples of A. **C**: SDS-PAGE analysis of various protein fractions collected during recombinant POSWOI purification. Lane 1: POSWOI transformant culture supernatant (Raw); Lane 2: POSWOI fraction collected after ultrafiltration (UF); Lane 3: POSWOI fraction collected after ammonium sulfate precipitation (SA); Lane 4: POSWOI fraction eluted in immobilized metal affinity chromatography (IMAC). Arrows show the recombinant POSWOI and native CBHI.

To obtain sufficient quantities of POSWOI protein, the recombinant expression of *poswo1* was driven by the strong inducible *T*. *reesei* cellobiohydrolase I (*cbh1*) promoter. In total, we obtained twenty transformants on selective PDA plates; six with intact *poswo1* coding sequences detected by PCR. Recombinant POSWOI can secrete into the culture medium and has a molecular weight (MW) of approximately 81 kD (Figure 2). Such a MW is much larger than the 50 kD weight calculated for POSWOI. In addition, another band with a small MW of 62 kD was also recognized by anti-his tag antibody, probably due to minor degradation of POSWOI under the denaturing conditions in SDS-PAGE.

Among the six *poswo1* transformants, transformant POS-20 had the highest POSWOI expression level of 105 mg/L. Interestingly, the expression of cellobiohydrolase I (CBHI) significantly decreased in transformants highly expressing POSWOI, such as transformants of POS-10 and POS-20. However, CBHI indicated a significant expression in POSWOI-less-expressed transformants, such as transformants of POS-12, POS-13, and POS-17 (Figure 2A), resembling that in wild-type *T*. *reesei* RUT-C30. The PCR assay revealed the integrity of *cbh1* cassette in the POSWOI-highly-expressed transformant POS-13 and POSWOI-less-expressed transformant POS-20 (see Additional file 1). The reason for the inverse expression tendency between CBHI and POSWOI needs further investigation.

With consecutive manipulation of ultrafiltration, ammonium sulfate precipitation, and immobilized-metal affinity chromatography (IMAC), recombinant POSWOI was successfully purified, obtaining a recovery of approximately 19% (Figure 2C).

### Disruptive activity of POSWOI on crystalline cellulose

**Figure 3:**
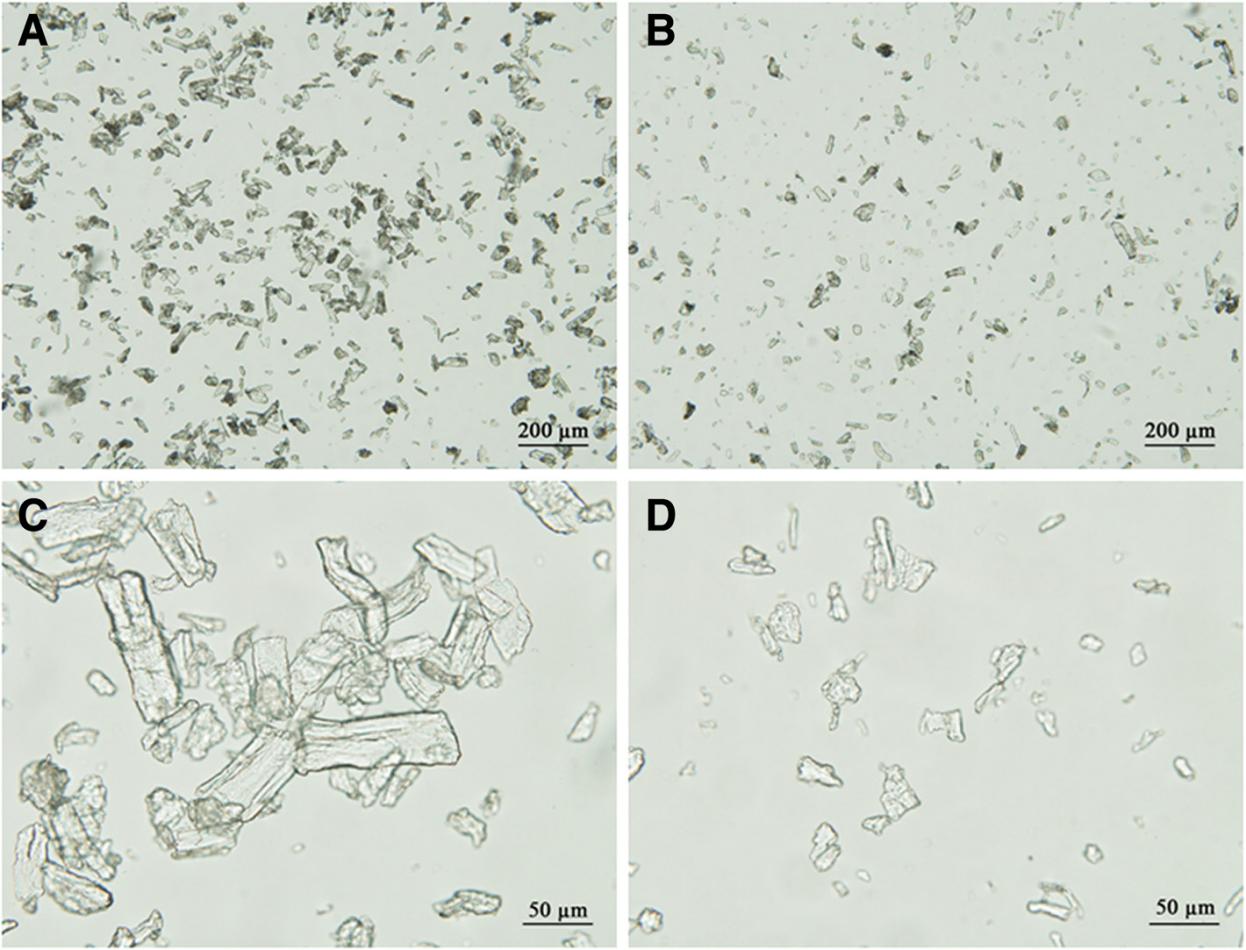
**Disruptive activity of POSWOI on Avicel.** 2.5 mg of Avicel was mixed without (**A**, **C**) or with (**B**, **D**) 10 μg of POSWOI at 50°C for 48 h in 250 μL 0.05 mol/L sodium acetate buffer, and then observed under light microscope.

**Figure 4:**
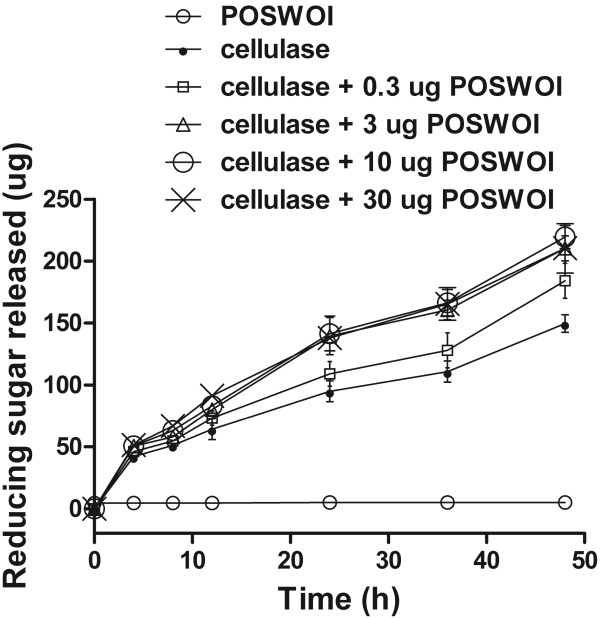
**Synergism between POSWOI and cellulases.** Different quantities of purified POSWOI (0.3, 3, 10, 30 μg) and a fixed quantity of cellulases (0.002 FPU) were incubated with 2.5 mg of Avicel in 250 μL of 0.05 mol/L sodium acetate buffer at 50°C. Samples were collected at 0, 4, 8, 12, 24, 36, 48 h, respectively, and subjected to reducing sugar evaluation by dinitrosalicylic acid (DNS) reagent. The reaction containing cellulases or POSWOI alone served as a control. Each reaction was performed in triplicate, and the data points and error bars indicate the mean ± standard deviations.

Swollenin has been demonstrated to be able to disrupt the structure of solid substrates, such as cotton fiber, filter paper, and plant cell walls [10,24]. We incubated POSWOI with Avicel and observed the disruptive activity of POSWOI on the substrate. Images under the light microscope showed that Avicel became smaller when treated with POSWOI (Figure 3B, D) compared with no POSWOI treatment (Figure 3A, C), indicating a disruptive activity of POSWOI on crystalline cellulose. There was no detectable reducing sugar in the reaction containing Avicel and POSWOI (Figure 4), suggesting that the disruptive effect of POSWOI on crystalline cellulose is not a hydrolytic process, similar to that of *T*. *reesei* swollenin [10].

### Hydrolysis of crystalline cellulose by POSWOI and cellulases

Conversion of crystalline regions within cellulose into less crystalline structures is an essential step for efficient biomass hydrolysis [5]. With a disruptive activity on the crystalline structure of cellulose, POSWOI might be capable of enhancing the cellulosic hydrolysis catalyzed by cellulases. We first tested whether there was synergism between POSWOI and cellulases in cellulosic saccharification. As Figure 4 showed, the Avicel incubated with both POSWOI and cellulases produced approximately 220 μg reducing sugars at 48 h, much more than the 150 μg that was produced by cellulases alone. The largest synergistic activity of POSWOI in this assay was 51.5% at 48 h. This showed that POSWOI can facilitate the saccharification of crystalline cellulose, and that there is a synergy between POSWOI and cellulases (Figure 4).

However, the synergy did not show linearity between the amount of POSWOI and the synergistic activity. When 0.3 μg and 3 μg POSWOI was used in the reaction, the synergistic activity at 48 h was 28.6% and 42.9%, respectively. Nevertheless, when the amount of POSWOI increased to 3 μg and 10 μg, the synergistic activity merely increased to 51.5% and 47.4%, respectively. These data suggest that POSWOI can become saturated, and that excessive POSWOI may even inhibit the interaction between POSWOI and cellulases.

**Figure 5:**
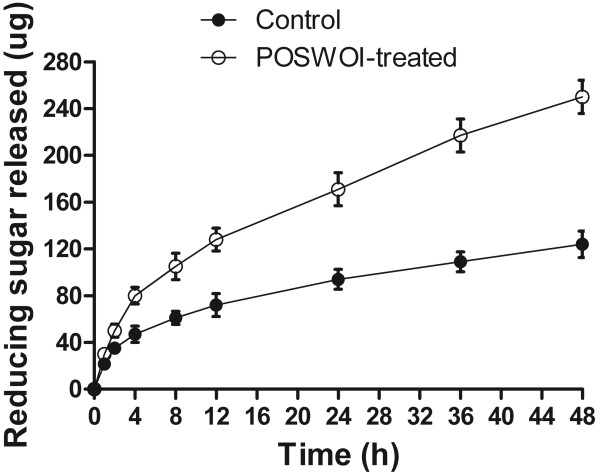
**Crystalline cellulose hydrolysis by cellulases with a POSWOI-pretreated procedure.** Purified POSWOI (10 μg) was first incubated with 2.5 mg of Avicel at 50°C for 48 h. The mixture was incubated at 90°C for 10 min to inactivate the POSWOI, and then rinsed with sodium acetate buffer to remove the POSWOI. Cellulases (0.002 FPU) were mixed with the pretreated Avicel in 250 μL 0.05 mol/L sodium acetate buffer at 50°C. Samples were collected at 0, 4, 8, 12, 24, 36, 48 h, respectively and subjected to reducing sugar evaluation by dinitrosalicylic acid (DNS) reagent. The Avicel pretreated by sodium acetate buffer only was used as a control. Each reaction was performed in triplicate, and data points and error bars indicate the mean ± standard deviations.

In addition to the simultaneous reaction, a two-step reaction was also performed to promote enzymatic saccharification. In the first step, Avicel was incubated with POSWOI alone, in the same manner as the disruptive activity assay for POSWOI. In the second step, POSWOI was removed from Avicel to minimize the binding competition of the substrate, and the pretreated Avicel was subjected to hydrolysis by cellulases alone. This reaction using POSWOI pretreatment produced 246 μg of reducing sugars at 48 h; nearly twice as many as the 124 μg produced by the acetate buffer control (Figure 5). This suggests that POSWOI better facilitates the enzymatic hydrolysis of crystalline cellulose when using a pretreatment procedure.

### Cellulase binding on POSWOI-treated crystalline cellulose

**Figure 6:**
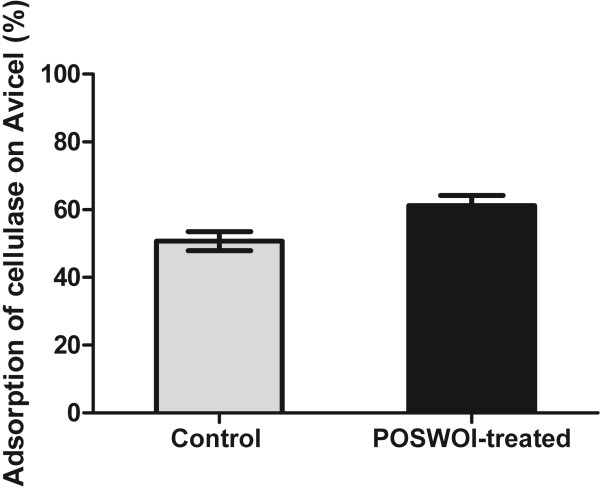
**Cellulase binding assay on POSWOI-pretreated Avicel.** 2.5 mg of Avicel was mixed with 10 μg of POSWOI at 50°C for 48 h. The mixture was then incubated at 90°C for 10 min to inactivate the POSWOI and rinsed with sodium acetate buffer to remove the POSWOI. The Avicel was then incubated with 0.08 FPU cellulases at 4°C for 2 h with rotation. The amount of cellulase bound on the POSWOI-pretreated Avicel was calculated by subtracting the quantity of cellulases in the supernatant from the total cellulases added in the reaction. The cellulases bound on the sodium acetate buffer-pretreated Avicel were used as a control. Each reaction was performed in triplicate, and the data points and error bars indicate the means ± standard deviations.

A cellulase binding assay was performed to reveal the mechanism of POSWOI pretreatment on promotion of saccharification. An experiment was carried out resembling the two-step procedure except that Avicel incubated with cellulases was placed at 4°C in order to minimize the cellulose hydrolysis. Only 50.7% of total cellulases bound to the Avicel without POSWOI pretreatment. However, 61.2% of the total cellulases could bind to the Avicel that was pretreated with POSWOI, suggesting a promotion of cellulase adsorption by the pretreatment (Figure 6).

## Discussion

Plant expansins have been well investigated for their relaxation activity on plant cell walls. It is believed that expansins can break down the hydrogen bonds between cellulose microfibrils without hydrolyzing them [8,9]. Swollenin, the newly reported protein in filamentous fungi, has sequence and functional similarity to plant expansins [10]. The structure of crystalline cellulose is highly ordered and maintained mainly by a great number of intra- and intermolecular hydrogen bonds. Therefore, the use of expansins or swollenin to enhance lignocellulosic hydrolysis is a promising application in the biofuel industry. Baker et al. [25], first used β-expansin from Zea mays in the hydrolytic reaction of lignocellulose and showed that β-expansin can improve the saccharification rate over the range of 55-80% conversion. Cucumber α-expansin was also able to enhance the hydrolysis of CMC-Na and high-esterified pectin by endoglucanase Cel 12A and pectinase, respectively [26]. Swollenin was used in a chimeric enzyme, in which swollenin was fused upstream of feruloyl esterase A, and increased the yield of ferulic acid by 50% [14]. Recently, an Aspergillus fumigatus swollenin Swo1 enhanced the saccharification rate of Avicel by 20% (from 61.2% to 74.4%) [24]. In our study, POSWOI increased the hydrolysis of crystalline cellulose Avicel by approximately 50%.

However, a saturation effect might exist during the reaction of POSWOI. POSWOI has a CBM that is also present in many cellulases. POSWOI and cellulases may thus compete for the limited binding sites in crystalline cellulose. This is probably the reason why POSWOI showed a saturation effect during cellulose hydrolysis, particularly when larger quantities of POSWOI were used. A POSWOI pretreatment was used to overcome the potential competition between POSWOI and cellulases on solid substrate. We obtained a nearly 100% improvement in Avicel saccharification. Recently, a similar study of swollenin pretreatment has also been reported, in which Kluyveromyces lactis-produced swollenin enhanced the hydrolysis of cellulosic substrates [15].

Several factors including the crystallinity, degree of polymerization (DP), accessibility, particle size, and pore volume, can influence the enzymatic hydrolysis rate of crystalline cellulose [27,28]. We found that POSWOI was able to disrupt the crystalline cellulose Avicel into smaller particles, significantly increasing the surface area and the accessibility of the substrate, and therefore enhancing the adsorption of cellulase from the substrate. The cellulase binding assay showed that cellulase did show an increased adsorption on POSWOI-pretreated Avicel.

Obtaining sufficient amount of active protein is a prerequisite for the study of gene function. One method to increase the yield of recombinant protein is to use a strong promoter. The *T*. *reesei* cellobiohydrolase I (*cbh1*) promoter is considered to be one of the strongest promoters in *T*. *reesei*[29,30]. We found that *cbh1* promoter successfully drove the expression of *poswo1*. Similarly, *T*. *pseudokoningii* swollenin was efficiently produced using the *cbh1* promoter [11].

Whether there is a relationship between swollenin and CBHI is not known. We found that CBHI was significantly down-regulated when POSWOI was highly expressed; however, CBHI normally expressed in the transformants that produced relatively low levels of POSWOI. As the C1-Cx hypothesis introduced, saprophytic microbes synthesize two classes of components, including a non-hydrolytic component C1 and endo- or exo-acting cellulases, together termed Cx [31,32]. Swollenin is a good candidate for component C1 due to its distinct disruptive activity on crystalline cellulose [10]. POSWOI displayed a considerable disruptive activity and was capable of enhancing the hydrolysis of cellulose by cellulases. Therefore, fewer cellulases including CBHI might be competent to produce sufficient living materials for fungal growth when large quantities of POSWOI are present. Actually, the reducing sugar in the culture supernatant of the POSWOI-highly-expressed transformant, POSWOI-less-expressed transformant, and the parental strain RUT-C30 was measured on the fifth day during cellulose induction. No significant difference among these strains in the amount of reducing sugars was observed (see Additional file 2), suggesting a facilitating effect of POSWOI on cellulosic hydrolysis.

## Conclusions

A novel gene *poswo1* (GeneBank No. HQ291307) with sequence similarity to *T*. *reesei* swollenins was isolated from the filamentous fungus *Penicillium oxalicum*. Recombinant POSWOI was produced at a high level in *T*. *reesei* and revealed disruptive activity on crystalline cellulose. With simultaneous and two-step procedures, POSWOI can significantly enhance the hydrolysis of crystalline cellulose, providing a new method for the efficient bioconversion of biomass.

## Methods

### Cloning of the *poswo1* gene

*Penicillium oxalicum* (ATCC 62501) genomic DNA was isolated using the CTAB method [33]. The conserved fragment of *P*. *oxalicum* swollenin gene was amplified using the degenerate primers 5’-THGTRGACAGCTGYCC-3’ and 5’-CGRCCCATGGCAATRTC-3’. The 3’ and 5’ flanking sequences of the swollenin conserved fragment were isolated using Genome Walking Kit (TaKaRa) based on the Thermal Asymmetric Interlaced PCR (TAIL-PCR) method [34]. Finally, the full-length coding sequence of *P*. *oxalicum* swollenin, termed *poswo1*, was amplified from *P*. *oxalicum* genomic DNA.

### Expression vector construction and transformation

To construct the *poswo1* expression vector, the coding sequence of *poswo1* omitting its own signal sequence was amplified from *P*. *oxalicum* genomic DNA using the primers Po-F1 5’-ATCGGCCTTCTTGGCCACAGCTCGTGCTCAGTCGGCCTGCCACCACCATCAC-3’ and Po-R1 5’-ACTATGCGGCCGCCTAAGTGAACTGCACTCCCAGGTCG-3’. The PCR product was then used as template for another round of PCR using the primers Po-F2 5’-CAGTCGGCCTGCCACCACCATCACCATCACCAATCCTCCTGTGCCTCTGCCTGG-3’ and Po-R1. The final PCR product was digested with Sfi I and Not I, and inserted into the pPIC-PST vector between Sfi I and Not I sites to produce pPIC-*poswo1*. The pPIC-PST vector consisted of *T*. *reesei* cellobiohydrolase I (*cbh1*) promoter, signal sequence, terminator, and a zeocin-resistance gene. The pPIC-*poswo1* was then co-transformed with pAN7-1, a hygromycin-resistant gene-containing vector, into the protoplasts of *T*. *reesei* RUT-C30 (ATCC 56765) [35]. The transformants were selected on potato dextrose agar (PDA) (ATCC medium 336) supplemented with 50 μg/mL hygromycin and purified to uninuclear clone by diluting spores on PDA plates. The *cbh1* expression cassette in *poswo1* transformants was assayed by PCR using the primers CBHI-F: 5’-CAGCGTACCCGTACAAGTCGTAATC-3’ and CBHI-R: 5’- TGGTACTGGGATACACGAAGAGCG-3’. The coding sequence (CDS) of *poswo1* in transformants was amplified using the primers Po-F1 and Po-R1.

### Production and purification of recombinant POSWOI

The spores of POSWOI-producing transformants were cultivated in minimal medium (MM) supplemented with 0.3% glucose, 0.2% peptone, 3% Avicel (Sigma PH101) at 28°C for 5 d with shaking at 250 rpm. The MM contained 0.14% (NH_4_)_2_SO_4_, 0.3% urea, 0.2% KH_2_PO_4_, 0.03% CaCl_2_, 0.03% MgSO_4_•7H_2_O, 0.0005% FeSO_4_•7H_2_O, 0.00017% ZnSO_4_•7H_2_O, 0.0002% CoCl_2_, 0.00016% MnSO_4_•H_2_O (w/v), and 0.05% Tween-80. The reducing sugar produced in the culture supernatant was estimated using the dinitrosalicylic acid (DNS) reagent. A 1.5 mL of the culture supernatant was mixed with 3.0 mL DNS reagent and placed in boiling water for 5 min. The absorbance of the supernatant at 540 nm was measured and the reducing sugar released was determined by reference to a glucose standard curve.

The culture supernatant of POSWOI transformant was 10-fold concentrated by a tangential flow ultrafiltration unit with a molecular weight cutoff of 10,000 (Vivaflow 50; Sartorius). The protein pellet precipitated from the ultrafiltrate with ammonium sulfate in 70% saturation was dissolved in binding buffer (0.05 mol/L Tris-HCl, 0.5 mol/L NaCl, pH8.0) and loaded on a Chelating Sepharose Fast Flow column charged with Ni^2+^ ions (GE Healthcare). The column was eluted with a linear imidazol gradient (0 ~ 0.5 mol/L imidazol in 0.05 mol/L Tris-HCl, 0.5 mol/L NaCl, pH8.0). The POSWOI-rich fraction was then desalted using HiTrap desalting column (GE Healthcare) and changed to 0.05 mol/L sodium acetate buffer (pH 4.8). The purified recombinant POSWOI was quantified with Bradford Protein Assay Kit (Bio Basic Inc) and verified using mouse monoclonal anti-his tag antibody (ABGENT) followed by a secondary antibody-alkaline phosphatase conjugate (Proteintech Group).

### Disruptive activity of POSWOI and cellulase binding assay

For disruptive activity assay, purified POSWOI (10 μg) was incubated with 2.5 mg crystalline cellulose Avicel in 250 μL 0.05 mol/L sodium acetate buffer (pH 4.8) at 50°C for 48 h in a rotating oven (Labnet, ProBlot 12S) at 60 rpm. The structure of Avicel was then observed under light microscope (Olympus BX51). For the cellulase binding assay, the Avicel was first treated as in the disruptive activity assay. The mixture of POSWOI and Avicel was then incubated at 90°C for 10 min to inactivate the POSWOI. The supernatant of the mixture was removed after brief centrifugation. The Avicel pellet was washed twice with sodium acetate buffer and incubated with 0.08 FPU cellulases under rotation at 4°C for 2 h. The cellulases bound on the POSWOI-pretreated avecil were calculated by subtracting the quantity of cellulases in the supernatant from the total added to the mixture. The cellulases bound on the sodium acetate buffer-pretreated avecil were used as a control.

### Enzymatic saccharification of crystalline cellulose

(1) Synergism between cellulases and POSWOI. Various quantities of purified POSWOI (0.3, 3, 10, 30 μg) were incubated with constant amounts of 0.002 filter paper cellulase units (FPU) in 250 μL 0.05 mol/L sodium acetate buffer (pH 4.8) containing 2.5 mg Avicel, respectively. The cellulases were the culture supernatant of the wild-type *T*. *reesei* RUT-C30 under the same growth conditions as POSWOI-producing transformants. The reaction was incubated at 50°C in a rotating oven at 60 rpm. The reaction containing cellulases or POSWOI alone served as a control. Samples were collected at 0, 4, 8, 12, 24, 36, 48 h, respectively and the reducing sugar released was evaluated. (2) POSWOI pretreatment and subsequent cellulosic hydrolysis by cellulases. Purified POSWOI (10 μg) was incubated with 2.5 mg Avicel in 250 μL 0.05 mol/L sodium acetate buffer (pH 4.8) at 50°C for 48 h with rotation. The mixture was incubated at 90°C for 10 min followed by a brief centrifugation. The supernatant of the mixture was removed and the Avicel pellet was washed twice with sodium acetate buffer. The POSWOI-pretreated Avicel was then mixed with cellulases (0.002 FPU) in 250 μL of sodium acetate buffer followed by rotation at 50°C. Samples were collected at 0, 4, 8, 12, 24, 36, 48 h, respectively, and the reducing sugar released was analyzed using the DNS method. Avicel pretreated by sodium acetate buffer only was used as a control. Each reaction was performed in triplicate and the data were expressed as means ± standard deviations. Synergistic activity was calculated as follows:

Synergistic activity (%) = (total reducing sugar released by both cellulases and POSWOI / reducing sugar released by cellulases alone -1) × 100% [12].

## Abbreviations

bp: Base pair; CBHI: Cellobiohydrolase I; CMC-Na: Sodium carboxymethyl cellulose; d: Day; DP: Degree of polymerization; DNS: Dinitrosalicylic acid; FPU: Filter paper units; IMAC: Immobilized metal affinity chromatography; kD: Kilodalton; LB: Luria-bertani; min: Minute; MM: Minimal medium; MW: Molecular weight; PDA: Potato dextrose agar; rpm: Round per minute; TAIL-PCR: Thermal asymmetric interlaced PCR.

## Competing interests

The authors declare that they have no competing interests.

## Authors’ contributions

KK performed the experiments, analyzed the data, and wrote the manuscript. SW revised the manuscript. GL(ai) carried out the gene cloning. GL(iu) and MX designed the study, and finalized the manuscript. All authors read and approved the final version.

## Supplementary Material

Additional file 1: Figure S1 The PCR assay of *cbh1* cassette in the genomic DNA of *poswo1* transformants. Figure showed the integrity of *cbh1* expression cassette in POSWOI-less-expressed transformant POS-13 and POSWOI-highly-expressed transformant POS-20. Click here for file

Additional file 2: Figure S2 The reducing sugar assessment of the culture supernatant of different *poswo1* transformants. Figure showed the amount of reducing sugar in the culture supernatant of the parental strain RUT-C30, POSWOI-less-expressed transformant POS-13, and POSWOI-highly-expressed transformant POS-20 on the fifth day during cellulose induction. Click here for file
