# Supplementary material for: Characterization of a novel swollenin from Penicillium oxalicum in facilitating enzymatic saccharification of cellulose
Source: BMC Biotechnol. 2013 May 20;13:42. doi: 10.1186/1472-6750-13-42 (PMC3681723; doi:10.1186/1472-6750-13-42)
Supplement: Additional file 1: Figure S1 — The PCR assay of cbh1 cassette in the genomic DNA of poswo1 transformants. Figure showed the integrity of cbh1 expression cassette in POSWOI-less-expressed transformant POS-13 and POSWOI-highly-expressed transformant POS-20. [file 1472-6750-13-42-S1.doc]

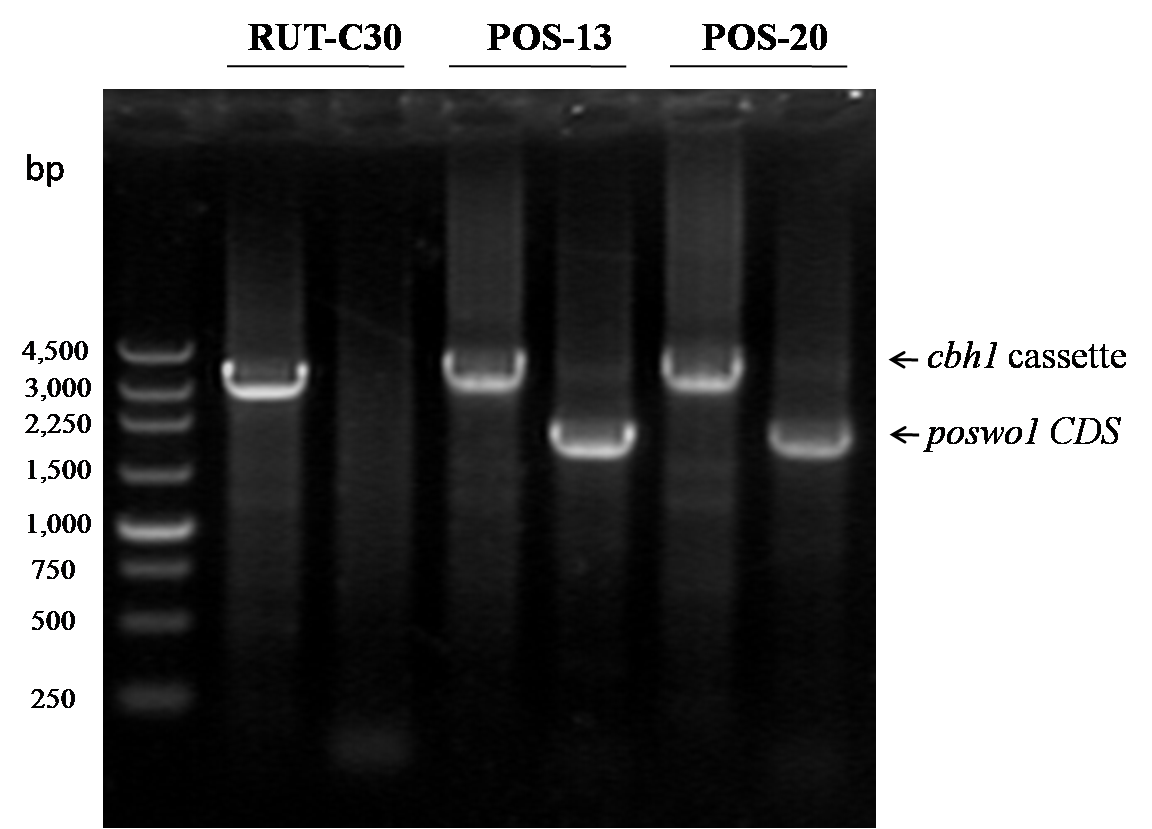


**Additional file 1:** **Supplementary Figure 1: the PCR assay of *cbh1* cassette in the genomic DNA of *poswo1* transformants**. Figure showed the *cbh1* expression cassette (~3,100 bp band) in POSWOI-less-expressed transformant POS-13 and POSWOI-highly-expressed transformant POS-20. The coding sequence (CDS) of *poswo1* (~1,900 bp band) was also assayed in the genomic DNA of transformants.
