# Supplementary material for: Characterization of a novel swollenin from Penicillium oxalicum in facilitating enzymatic saccharification of cellulose
Source: BMC Biotechnol. 2013 May 20;13:42. doi: 10.1186/1472-6750-13-42 (PMC3681723; doi:10.1186/1472-6750-13-42)
Supplement: Additional file 2: Figure S2 — The reducing sugar assessment of the culture supernatant of different poswo1 transformants. Figure showed the amount of reducing sugar in the culture supernatant of the parental strain RUT-C30, POSWOI-less-expressed transformant POS-13, and POSWOI-highly-expressed transformant POS-20 on the fifth day during cellulose induction. [file 1472-6750-13-42-S2.doc]

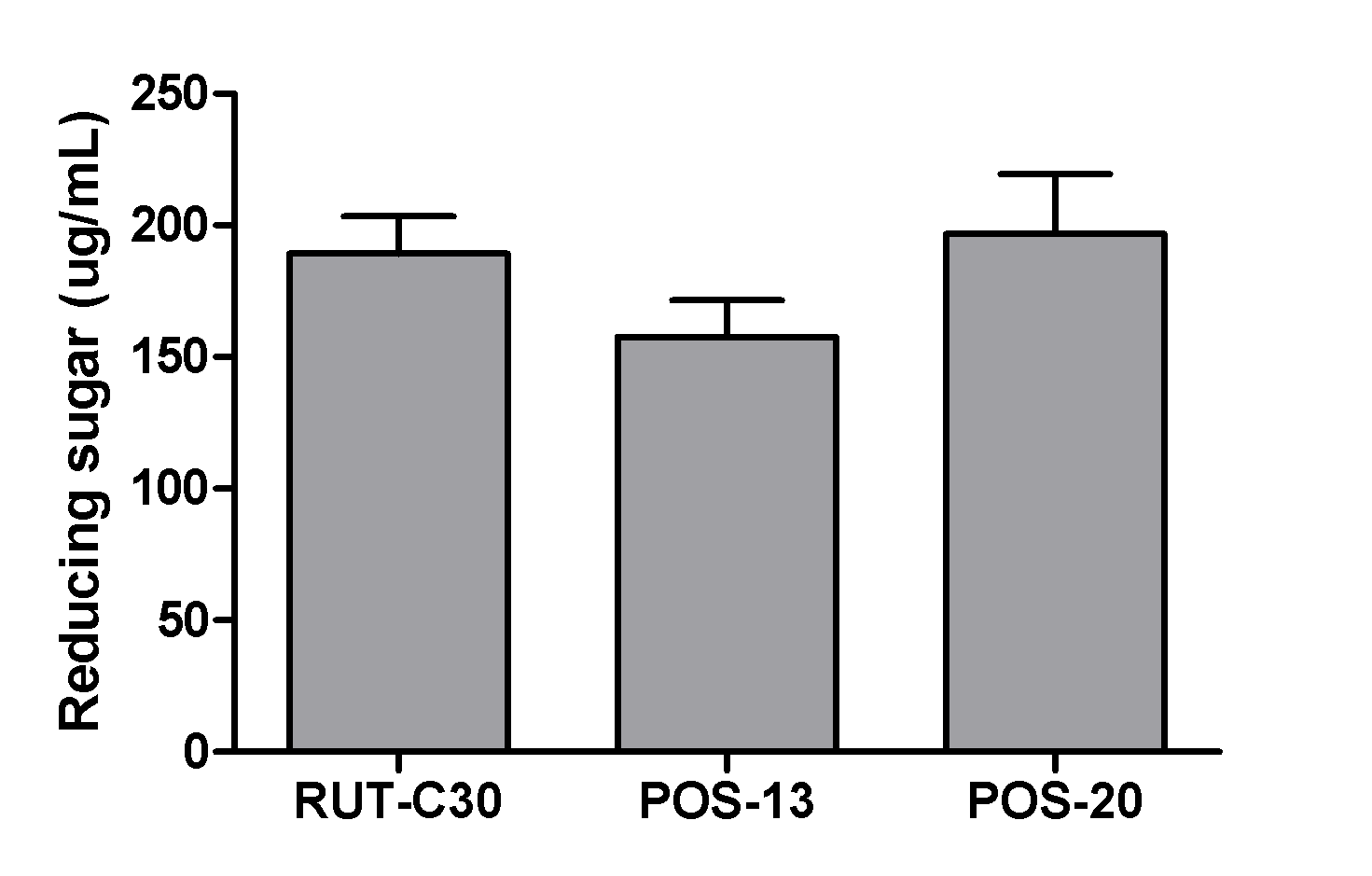


**Additional file 2:** **Supplementary Figure 2: the reducing sugar assessment of the culture supernatant of different *poswo1* transformants**. Figure showed the amount of reducing sugar in the culture supernatant of the parental strain RUT-C30, POSWOI-less-expressed transformant POS-13, and POSWOI-highly-expressed transformant POS-20 on the fifth day during cellulose (3% Avicel) induction. The reducing sugar produced in the culture supernatant was estimated using the dinitrosalicylic acid (DNS) reagent. A 1.5 mL of the culture supernatant was mixed with 3.0 mL DNS reagent and placed in boiling water for 5 min. The absorbance of the supernatant at 540 nm was measured and the reducing sugar released was determined by reference to a glucose standard curve.
